# Global core indicators for measuring WHO’s paediatric quality-of-care standards in health facilities: development and expert consensus

**DOI:** 10.1186/s12913-022-08234-5

**Published:** 2022-07-08

**Authors:** Moise Muzigaba, Tamar Chitashvili, Allysha Choudhury, Wilson M. Were, Theresa Diaz, Kathleen L. Strong, Debra Jackson, Jennifer Requejo, Anne Detjen, Emma Sacks

**Affiliations:** 1 Department of Maternal, Newborn, Child, and Adolescent Health, and Ageing, World Health Organization, Avenue Appia 20, 1202 Geneva, Switzerland; 2 University Research Co. LLC, 4600 Creek Shore Dr. Rockville, Rocville, MD 20852 USA; 3 Department of Maternal and Child Health, Gillings School of Global Public Health, University of North Carolina, 135 Dauer Dr, Chapel Hill, Chapel Hill, NC 27599 USA; 4 Data & Analytics Section, UNICEF, 3 UN Plaza, New York, NY 10017 USA; 5 London School of Hygiene and Tropical Medicine, Faculty of Epidemiology and Population Health, Department of Infectious Disease Epidemiology, Keppel Street, London, UK; 6 School of Public Health, University of the Western Cape, Bellville, PBX17 South Africa; 7 Child and Community Health Unit, UNICEF, 3 UN Plaza, New York, NY 10017 USA; 8 Department of International Health, Johns Hopkins School of Public Health, 615 North Wolfe St, Baltimore, MD USA

**Keywords:** Quality-of-care, Child health, Young adolescent, Robust, Measurement, Indicators, Methodology, Global, Consultation, WHO quality-of-care standards

## Abstract

**Background:**

There are currently no global recommendations on a parsimonious and robust set of indicators that can be measured routinely or periodically to monitor quality of hospital care for children and young adolescents. We describe a systematic methodology used to prioritize and define a core set of such indicators and their metadata for progress tracking, accountability, learning and improvement, at facility, (sub) national, national, and global levels.

**Methods:**

We used a deductive methodology which involved the use of the World Health Organization *Standards for improving the quality-of-care for children and young adolescents in health facilities* as the organizing framework for indicator development. The entire process involved 9 complementary steps which included: a rapid literature review of available evidence, the application of a peer-reviewed systematic algorithm for indicator systematization and prioritization, and multiple iterative expert consultations to establish consensus on the proposed indicators and their metadata.

**Results:**

We derived a robust set of 25 core indicators and their metadata, representing all 8 World Health Organization quality standards, 40 quality statements and 520 quality measures. Most of these indicators are process-related (64%) and 20% are outcome/impact indicators. A large proportion (84%) of indicators were proposed for measurement at both outpatient and inpatient levels. By virtue of being a parsimonious set and given the stringent criteria for prioritizing indicators with “quality measurement” attributes, the recommended set is not evenly distributed across the 8 quality standards.

**Conclusions:**

To support ongoing global and national initiatives around paediatric quality-of-care programming at country level, the recommended indicators can be adopted using a tiered approach that considers indicator measurability in the short-, medium-, and long-terms, within the context of the country’s health information system readiness and maturity. However, there is a need for further research to assess the feasibility of implementing these indicators across contexts, and the need for their validation for global common reporting.

**Supplementary Information:**

The online version contains supplementary material available at 10.1186/s12913-022-08234-5.

## Background

Globally, 60% of preventable deaths are due to poor-quality care, and it has been estimated that one out of three patients across low- and middle-income countries (LMICs) report suboptimal client-centred care [1, 2]. Although coverage of lifesaving interventions for many priority health conditions—including child health—has improved globally, this has not consistently translated into survival for preventable health conditions [3]. Providing health services without guaranteeing quality is ineffective, wasteful, and unethical [2].

**Fig. 1:**
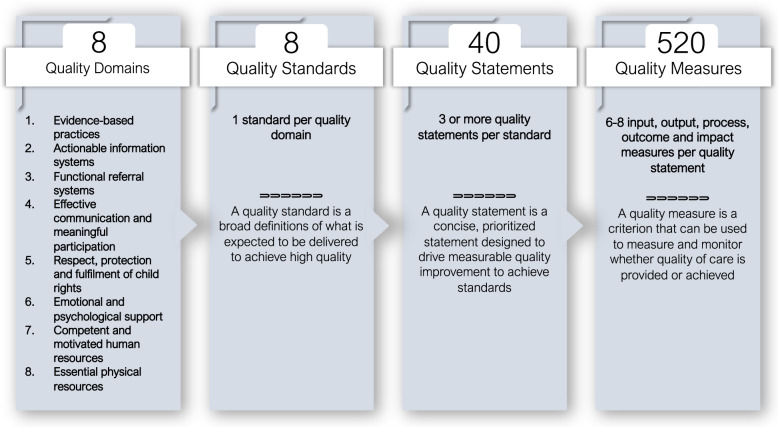
Structure of the WHO QSs for improving the quality of paediatric and young adolescent care in health facilities

In 2015, the World Health Organization (WHO) and partners articulated a vision in which “*Every woman, newborn, child and adolescent receives quality health services throughout the continuum of their life course and level of care*” [4]. To accompany this vision, in 2016, WHO developed Quality Standards (QSs) for Maternal and Newborn Health (MNH) in health facilities [5]. This was followed by the publication in 2018 of WHO’s Paediatric and Young Adolescent QSs (hereinafter referred to *paediatric QSs*) [6], and later, the WHO’s Small and Sick Newborn QSs published in 2020 [7]. All these QSs encompass both the provision and experience of care as key dimensions of quality and define eight domains of quality (Fig. 1) that should be assessed, improved, and monitored across health system levels [6–8].

There is currently a plethora of paediatric QoC indicators available in both the published and grey literature. However, there are yet to be global recommendations for a robust set of core indicators that can be measured routinely or periodically in health facilities to track and compare progress and drive improvement and accountability at every level of the health system [2, 8–10]. In this paper, we describe a systematic methodology we used to prioritize and define a robust set of core paediatric and young adolescent QoC indicators (hereinafter referred to as “*core indicators*”) to support global efforts around paediatric and young adolescent health service quality improvement, progress tracking, and accountability across the health system.

Given the current gaps in the standardization, availability, and comprehensiveness of paediatric and young adolescent health information in most LMICs, we aimed to develop core indicators that would measure different aspects of the quality standards, without regard to feasibility of measurement across health information systems. The proposed set of core indicators therefore contains tiers of indicators that can be measured either immediately or in future depending on the maturity and readiness of health information systems in different countries. As such, we also make a case for transformative efforts to reform national health information systems and technologies to accommodate more robust and essential quality indicators, as opposed to promoting the adoption and use of “convenient-to-measure” quality indicators. We argue that the proposed set of core indicators would allow for the measurement of critical input, process, outcome, and impact dimensions of care which can serve as high level “signals” of paediatric and young adolescent QoC in health facilities, while retaining utility at (sub)national, national, and global levels.

## Methods

### The organizing framework for indicator development

Figure 1 shows the structure of WHO’s paediatric QSs. The 8 QSs describe what should be provided to achieve high-quality health care for children and young adolescents across the 8 quality domains (QDs). The 40 corresponding quality statements (see Additional File 1) are designed to drive continuous improvement to achieve positive care outcomes and a positive experience of care. Five-hundred and twenty (520) quality measures (QMs) were identified from the 8 domains as a means to measure whether specific aspects of quality are provided or achieved.

The structure and content of the MNH QSs and Paediatric QSs were adopted in the maternal, newborn, and child health (MNCH) QoC monitoring framework as the *organizing framework* for developing core QoC indicators [11]. The MNCH QoC monitoring framework recognized the different QoC measurement needs by stakeholders across different levels of the health system and proposes three QoC measurement components, including a) *core (common) indicators*: which is a small set of prioritized input, process, outcome, and impact indicators for use by all stakeholders at every level of the health system to track and compare process across and within regions and countries; b) *Quality improvement* (*QI) indicator catalogue*: which consists of a flexible menu of indicators to support QI facility and subnational levels led—respectively—by facility-based QI teams and district or regional health authorities to support improving and sustaining QoC in health facilities; and c) *implementation milestones* which help track the progress of country-specific or subnational implementation activities to develop and maintain quality improvement programmes. However, the latter are not directly linked to QSs but rather to national QoC implementation roadmaps, policy and strategies.

Core indicators and the QI indicator catalogue are directly linked to and measure the QSs. The focus of this article is core indicators, and we describe how they were selected from the QI indicator catalogue to maintain the linkages with the QSs.

### Approach

**Fig. 2:**
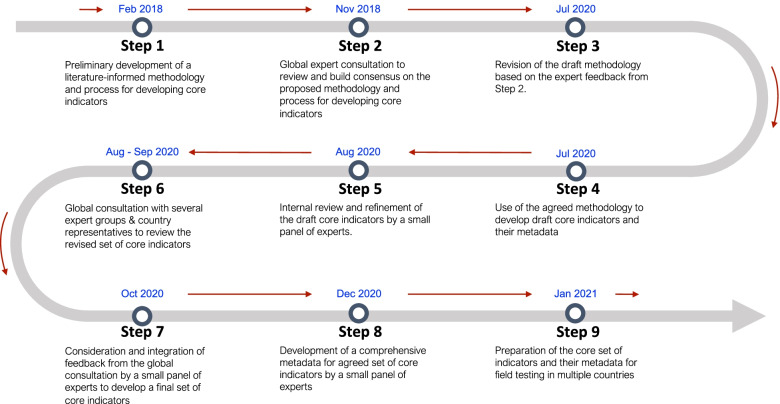
A stepwise process used to develop the core indicators

The proposed core indicators were developed using a deductive approach. This approach is typically built upon a specific conceptual framework and links the indicator to clinical and/or patient-centered inputs, processes, and outcomes [12]. The proposed core indicators were therefore linked to the specific QSs and QMs. To derive these indicators, we followed nine complementary steps (Fig. 2) which allowed for an iterative process of indicator development. These steps included a rapid literature review, development of a methodology, the application of a systematic algorithm for indicator systematization and prioritization, and multiple iterative expert consultations.

### Step 1

A gold standard methodology or approach for QoC indicator development is yet to be defined [13]. Therefore, we first conducted a rapid review of the published and grey literature to scope available methodological approaches for developing core QoC indicators. The most-commonly used approaches identified were: reviewing existing health information systems to identify and adopt or adapt already available QoC indicators [14], using guideline-based approaches to systematically align QoC indicators to specific clinical and non-clinical guidelines [15], deriving QoC indicators from the available evidence base (e.g., indicators that have been validated in the literature as good measures of quality), combining expert consensus with evidence review, or a combination of multiple approaches [13, 16]. Using a peer-review process, we analysed and synthesized the strengths and weaknesses of available approaches, their relevance to LMIC settings and used the results to develop a technical protocol that outlined our methodology for selecting core indicators.

### Steps 2&3

Following the development of the methodology document in step 1, WHO convened a global expert consultative meeting (Step 2a) to review and reach consensus on the proposed methodology and process for developing the quality indicators. Participants included 24 independent technical experts and 10 experts from WHO, USAID, and UNICEF that represented a range of expertise in paediatric QoC programming and measurement, paediatric research, health informatics, paediatric care, and child rights. During the consultation, experts were placed in small heterogenous groups based on their expertise to discuss and make recommendations on the methodology. Each group’s recommendations were presented in a plenary session for discussion and consensus building, and the resulting recommendations were presented in a separate consultative process (Step 2b) to the Child Health Accountability Tracking (CHAT) Technical Advisory Group [17]. The recommendations from the initial expert meeting and CHAT were all used to revise the methodology before implementation (Step 3).

### Step 4

**Fig. 3:**
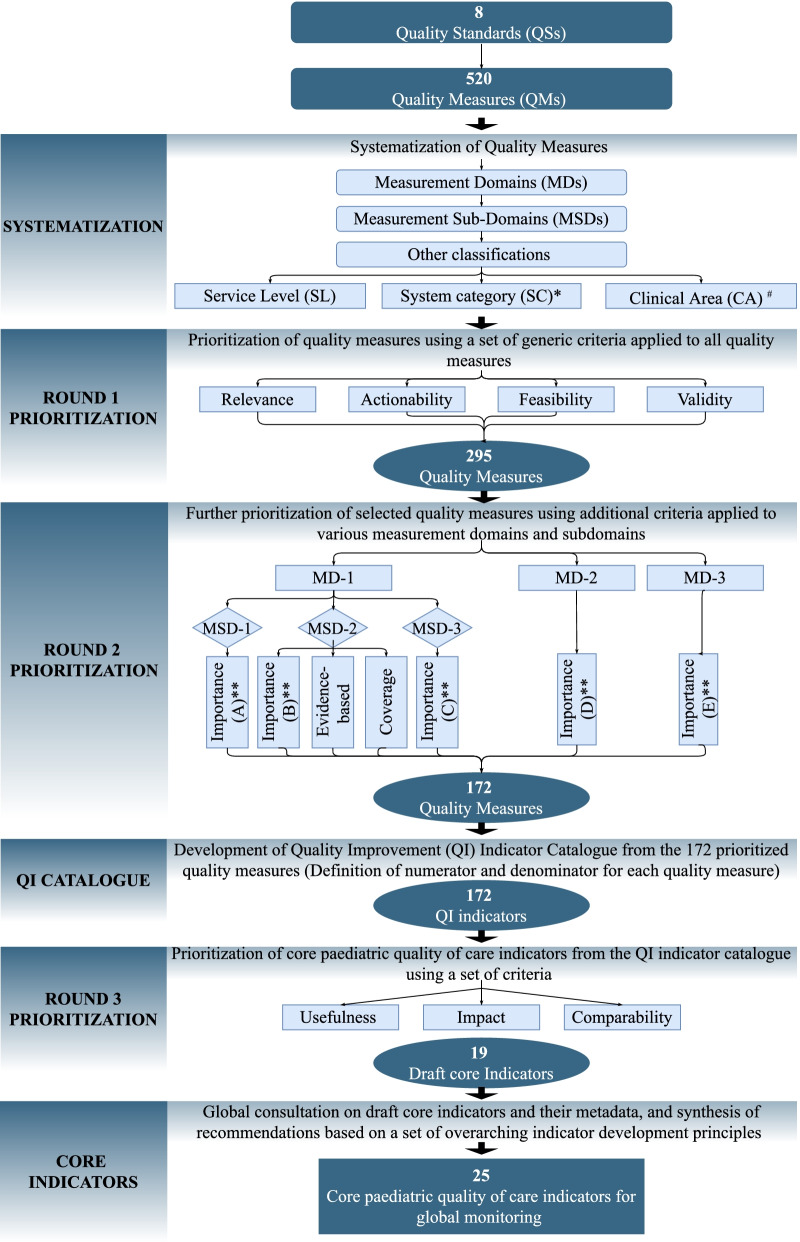
Flow diagram of core indicator prioritization and development process (*System categories: each QM was systematized by the QoC element it measured: input, process (adherence to EB practices, non-evidence-based, harmful practices), or a related outcome/impact. Input measures were further systematized by various input categories: a) Medicines, supplies and equipment, infrastructure; b) Clinical guidelines, protocols, job aides; c) Operational guidelines, protocols; d) trained human resources; e) availability of health services f) financing g) health information system; h) organization of care processes; i) oversight and management. **Importance*:* This criterion was used to prioritize all measurement subdomains, but, as shown in Table 1, it was framed differently under each measurement domain or subdomain. ^***#***^Clinical content area: List of prioritized clinical content areas used to select QMs is provided in the Additional File 3)

The expert panel recommended the use of indicator development criteria and principles (described later) that recognize the complexity of measuring quality of clinical care and patient or caregiver-reported experience of care. These principles guided the generation of the *most critical* input, process, output, outcome, and impact indicators that can be used as “*signals of quality*”, thereby eliminating the need for measuring every aspect of service provision and related outcomes. These principles were applied to the 520 QMs during the indicator development process using a criterion- and score-based indicator prioritization algorithm which we built in an interactive Microsoft Excel spreadsheet **(**see Additional File 2**)**. Initially, the algorithm allowed for the prioritization of a non-prescriptive menu of QMs linked to the QS. Based on this set, the core indicators were selected, identifying those which were most aligned with the QSs. The indicator prioritization algorithm is illustrated in Fig. 3.

### Development of QI indicator catalogue

As shown in Fig. 3, the QI indicator catalogue was generated from the 520 QMs. All QMs were first categorised into 3 measurement domains and 11 measurement subdomains as shown in Table 1. The objective of classifying QMs by measurement domains and measurement subdomains was to identify potential measurement areas to which distinct prioritization criteria should be applied. For example, the prioritization criterion for measurement domain 1 (adherence to evidence-based practices) aimed to identify areas for which there is strong evidence that links the care process to a desired health outcome. The same criterion, however, could not be applied to the QMs for child- and family-centered practices (measurement domain 3) which required consideration of patients’ rights, involvement in care, and elimination of harmful practices.

**Table 1:** Measurement domains and subdomains used to systematize QM

Measurement domains (MD)	Measurement sub-domains (MSD)
**MD-1:** Evidence-based (EB) practices for routine care of children and management of illness (*Standard 1*)	**MSD-1**: Inputs (clinical-content specific)
	**MSD-2**: Adherence to EB practices, and elimination of non-EB, harmful practices
	**MSD-3**: Care outcomes
**MD-2:** Cross-cutting supporting facility level health systems (*Standard 2, 3, 7 and 8*)	**MSD-4**: Actionable information systems (Standard 2)
	**MSD-5**: Functioning referral (Standard 3)
	**MSD-6**: Human resources (Standard 7)
	**MSD-7**: Physical resources (Standard 8)
**MD-3:** Child and family-centered practices/experience of care (*Standard 4, 5 and 6)*	**MSD-8**: Effective communication and meaningful participation (Standard 4)
	**MSD-9**: Respect, protection, and fulfilment of child rights (Standard 5)
	**MSD-10**: Emotional and psychological support (Standard 6)

Two categories of prioritization criteria were used: a) *Criteria applied to all QMs to prioritize valid, relevant, actionable, and feasible QMs*; and b) *Additional criteria applied to the resultant QMs in different measurement domains or subdomains to select QI indicators for the catalogue.* As described in Table 2, these criteria were built in a standardized Microsoft Excel template, which facilitated a systematic and sequential approach to selecting specific QMs based on a scoring algorithm (Additional File 2) developed using a Microsoft Excel visual basic application. The final set included only QMs that met the minimum cut-off score for both prioritization steps. The final QMs were assigned non-prescriptive numerators and denominators to constitute the *QI indicator catalogue* which formed the basis for prioritizing core indicators.

**Table 2:** Prioritization steps, criteria and scoring mechanism used to prioritize QI catalogue and core indicators

Prioritization step	Measurement domains	Measurement subdomains	Specific prioritization criteria and their application	Scoring mechanism to prioritize QMs
**Round 1: Using of a set of criteria applied to all measures**	**All**	**All**	The following criteria were applied to all QM from all the MD and their MSD • ***Relevance*:** Is QM specific to the QS of interest • ***Actionability***: Can the data collected for the measure guide clear and rapid QI actions and changes at the relevant health system level • ***Feasibility***: Are the data needed for the QM most likely available and accessible or can they be obtained without substantial resource investments (time, human and financial resources) – either now or in future • **Validity: **Can the measure truly measure what it purports to measure (face validity) •Reliability*: Are the results of the measure reproducible irrespective of who makes the measurement, from which data source or when it is made •Clarity/specificity*: Is the measure described in a clear and unambiguous terms	• Each of these criteria was scored using a 5-point scale with a minimum score of 1 and maximum score of 5. Thus, the minimum total score possible for each QM at this stage was 4 (1 minimum score X 4 criteria = 4), and the maximum score possible was 20 (5 maximum score X 4 criteria = 20). Using a predefined cut-off score of 16, which was the median of scores across all relevant QMs. Thus, a QM was considered for the next round of prioritization only if it had a score ≥ 16
**Round 2: Using additional criteria for specific MDs and MSDs to select Catalogue QMs**	**MD-1**	**MSD-1**	This criterion was only applied to QM under MSD-1 • **Importance (A) **How important is the input in delivering high impact evidence-based paediatric care intervention and achieve good care outcome?	•The impact criterion allowed for prioritization of various input measures for high impact clinical interventions. However, different types of input measures are not equally important for provision of evidence-based care. For example, availability of antibiotic for child with severe pneumonia may be more important for the care outcome than availability of operational guideline or job aid. To minimize subjectivity, different weights were applied to different types of inputs based on their relative importance in provision of evidence-based care • The minimum score per QM was 1, the maximum was 5. The cut-off score was set at 4, which was the median of all scores across all relevant QMs. A QM was prioritized further if it had a score ≥ 4
	**MD-1**	**MSD-2**	These criteria were only applied to QM under MSD-2 • ***Importance (B)****:* How much does the clinical condition/content area measured by the QM contribute to mortality or disease burden in specific settings • ***Strength of Evidence base:*** How strong is the evidence to link the clinical process to care outcome? • ***Coverage:*** How many children receive / could receive the clinical intervention that the QM measures	• The minimum score per QM was 3, and the maximum was 15 (maximum 5 score × 3 criteria = 15). The cut-off score was set at 13, which was the median of all scores across all relevant QMs. Thus, a QM was prioritized further if it had a score ≥ 13
	**MD-1**	**MSD-3**	This criterion was only applied to MSD-3 • ***Importance (C)****:* Considering that the criteria “coverage” and “impact” only apply to clinical interventions and are not relevant to care outcomes, “Importance” was the only criterion used to prioritize clinical outcomes	• The minimum score per QM was 1, and the maximum was 5. The cut-off score was set at 4 which was the median of all scores across relevant QMs. Thus, a QM was prioritized further it had a score ≥ 4
	**MD-2**	**MD-2**	This criterion was applied to all QM under MD-2 • ***Importance (D): ***How important specific cross-cutting facility level input is to improve care processes or health or family-centered outcomes?	• The minimum score per QM was 1, and the maximum was 5. The cut-off score was set at 4 which was the median of all scores across relevant QMs. Thus, a QM was prioritized further it had a score ≥ 4
	**MD-3**	**MD-3**	This criterion was applied to all QM under MD-3 • ***Importance (E):*** Does the corresponding standard support the following key principles: 1) the willingness and ability of patients and families to participate in care; 2) measures patient-reported outcome; 3) is built upon the principle of no harm and 4) patients’ right?	• Weights (scaled to 100%) were used to prioritize QM around child- and family-centered practices/experience of care: measures the ability of patients and families to participate in care (30%); measures patient reported outcome (20%); is built upon the principle of no harm (30%) and patients’ rights (20%) • The summary weighted score for each measure was then calculated. The minimum score for this domain was 1, the maximum was 5. The cut-off score was set at 2.5 which was the median of all scores across relevant QMs. Thus, a QM was prioritized further it had a score ≥ 2.5
**Round 3: Using additional criteria to select core indicators**	**All**	**All**	These criteria applied to all selected catalogue QM • **Usefulness: **does the measure focus of performance of the system at population level and once aggregated, is useful to different stakeholders to guide decisions and changes especially at national and global levels? • **Impact:** Is the measure sensitive to QoC interventions, assessing the highest impact of QoC intervention(s) to national and global child health priority (25) • **Comparability: **Is the measure aligned to the greatest extent possible with standardized and validated global childcare indicators/ monitoring frameworks and/or are comparable across countries and regions (26)	• Each of these criteria was scored using a 5-point scale with a minimum score of 1 and maximum score of 5 • The minimum score per each additional criteria was 1, and the maximum was 5. The cut-off score was set at 13 which was the median of all scores across relevant indicators. Thus, a catalogue indicator was prioritized as core it had a score ≥ 13

^*^ In addition to 4 criteria that were applied to all MDs and their MSDs, we scored each measure against two additional criteria (*reliability* and *clarity*). All measures were scored and color-coded using 1–5-point scale with a minimum score of 1 and maximum score of 5 against reliability and clarity (See additional file 2). To avoid losing important measures that were not fully defined at the initial stage, the scoring results of above two criteria were not included in the prioritization algorithm. The scoring results for reliability and clarity informed the indicator development process to further refine the definition and data collection methods of selected indicators

### Development of core indicators

The following criteria were applied to all indicators in the QI indicator catalogue to prioritize a smaller set of core indicators: usefulness, impact, and international comparability (see Table 2 for detailed rationale of each criterion). These criteria helped prioritise indicators for measuring QoC of paediatric services that are: 1) useful for guiding decisions around resource allocation and programming and especially at national and global levels; 2) sensitive to detecting change in QoC interventions at the service delivery level for priority paediatric conditions, and 3) aligned to the extent possible with standardized and validated global child health care indicators to enable comparisons.

These criteria and associated scores were applied to the QI indicator catalogue by a measurement expert (TC) who drafted a small set of core indicators for further review by two other experts (WW & MM). Details of the scoring mechanisms and cut-off scores are provided in Table 2.

### Steps 5–9

During Step 5, the draft set of core indicators generated from step 4 were reviewed internally by a small group of 3 experts from WHO and University Research Co (TC, WW, MM) to determine the extent to which they fulfilled the selection criteria. Once the review was completed, the draft core indicators went through a global expert consultation process (Step 6) to generate the feedback on the content, clarity, definition, prioritization and other elements of the indicator metadata. These included program officials responsible for child health in various WHO country offices and ministries of health across three WHO regional offices which participated (Regional office for Africa, Regional Office for South-East Asia, and the Regional Office for Europe); individual child health QoC programming and measurement experts from academic and research institutions; WHO’s implementing partners at country level; global technical working groups; as well as technical focal points from other UN and multilateral agencies. All the resulting recommendations from the review were synthesized and revised by a small panel of experts (TC, WW, MM), as part of Step 7, and integrated into the draft core indicators.

The WHO expert team made final decisions on the core indicator selection based on the following guiding principles derived from the literature and expert recommendations:

- ***Alignment with the QS and system categories*:** The recommended core indicator measures at least one QS.
- ***Focus on impact*:** The recommended core indicator can assess the clinical or QI interventions that would have the highest impact on child health or child and family-centered outcomes such as mortality, morbidity, respectful care, etc.
- ***Emphasis on child- and family-centered practices*:** The recommended core indicator can help to inform the development of interventions and practices that improve both child and family-centered care.
- ***Guiding QI actions at all levels*:** While collected from each health facility, aggregated data from the recommended core indicator can provide strategic and timely information to be used across all levels of the health system (district, region, national, global levels) for comparable analysis to guide decision-making and planning for QI.
- ***Provider or health system control*:** The recommended core indicator can measure attributes of service delivery and outcomes which are within the control of the health system or the provider.
- ***Sample size adequacy*:** The recommended core outcome and impact indicators should typically generate enough data that allow for subgroup analysis and statistical testing to explain whether the difference in performance levels is greater than what would be expected by chance.
- ***Relationship with quality*:** For recommended input and process indicators, there is sufficient evidence or reasonable assumption on their correlation with the outcome(s) of interest, even when there is no sufficient evidence on context-specificity or summative effects of these inputs and processes on the outcomes of interest

During step 8 the small expert group (TC, WW, MM) developed a comprehensive metadata and indicator dictionary for each recommended core indicator. Finally, (Step 9) these indicators and metadata were prepared for eventual field testing across different settings.

## Results

In Round 1 of the prioritization process, 295 quality measures were pre-selected. The review and prioritization of these measures in Round 2 resulted in a smaller set of 172 catalogue measures (Fig. 3). Initially, from Round 3, all 172 measures were retained and defined to constitute the QI indicator catalogue (not the focus of this paper). Finally, Round 3 generated 19 core indicators which, following the final expert consultation, were increased to 25 core indicators (Tables 3 & 4). Six more indicators were recommended to account for the respectful and experience of care components.

**Table 3:** Distribution of core indicators by MD, QS, indicator classification, and service level

**Measurement domains & Standards**	**Classification**						**Service level**							
	**Input**		**Process**		**Outcome / Impact**		**Inpatient**		**Outpatient**		**Both**		**Total**	
	n	(%)	n	(%)	n	(%)	n	(%)	n	(%)	n	(%)	n	(%)
**MD-1:** Evidence-based practices for routine care of children and management of illness	0	(0)	11	(44)	3*	(12)	2*	(8)	1	(4)	11	(44)	14	(56)
Standard 1: Evidence-based practices for routine care of children and management of illnesses	0	(0)	11	(44)	2	(8)	1	(4)	1	(4)	11	(44)	13	(52)
**MD-2:** Cross-cutting supporting facility level health systems	3	(12)	1	(4)	0	(0)	0	(0)	0	(0)	4	(16)	4	(16)
Standard 2: Actionable information systems	1	(4)	1	(4)	0	(0)	0	(0)	0	(0)	2	(8)	2	(8)
Standard 3: Functioning referral systems	0	(0)	0	(0)	0	(0)	0	(0)	0	(0)	0	(0)	0	(0)
Standard 7: Competent, motivated, empathetic human resources	1	(4)	0	(0)	0	(0)	0	(0)	0	(0)	1	(4)	1	(4)
Standard 8: Essential child and adolescent-friendly physical resources	1	(4)	0	(0)	0	(0)	0	(0)	0	(0)	1	(4)	1	(4)
**MD-3**: Child and family-centered practices/experience of care	1	(4)	4	(16)	2	(8)	1	(4)	0	(0)	6	(24)	7	(28)
Standard 4: Effective communication and meaningful participation	0	(0)	2	(8)	1	(4)	0	(0)	0	(0)	3	(12)	3	(12)
Standard 5: Respect, protection, and fulfilment of child rights	0	(0)	1	(4)	1	(4)	0	(0)	0	(0)	2	(8)	2	(8)
Standard 6: Emotional and psychological support	1	(4)	1	(4)	0	(0)	1	(4)	0	(0)	1	(4)	2	(8)
**Total # of core indicators**	**4**	**(16)**	**16**	**(64)**	**5**	**(20)**	**3**	**(12)**	**1**	**(4)**	**21**	**(84)**	**25**	**(100)**

*The core indicator of ****institutional child mortality*** (*) is broadly applicable across measurement domains and standards as an impact indicator but has been included in measurement domain 1 of evidence-based practices for simplicity of data presentation. Percentages displayed are percent of total core indicators (25)

**Table 4:** List of recommended core indicators and their definitions

Indicator name	Indicator definition	Indicator classification	Service level for measurement	Numerator	Denominator	Proposed disaggregation	Proposed data source	Proposed measurement method	Proposed measurement frequency
1.Institutional Child Mortality Rate	# of pre-discharge child deaths per 1000 children who visited the health facility	Impact	Inpatient	# of children who died in the health facility before discharge *(Includes deaths in the emergency ward but does not include children who died upon arrival at the hospital, child deaths during outpatient visits, and institutional neonatal deaths)*	# of children who visited the health facility for medical care during reporting period	•Major causes of death •Sex •Types of inpatient facilities •Age groups (0–7 days, 8–27, 28–59 days, 60 days- < 1 year, 1- < 5 y, 5- < 10, 10- < 15 y) •Death within and after 24 h of admission	Routine HMIS^a^	Review of paediatric ward register or patient medical records, paediatric death audit and triangulation of information if possible	Monthly
2.In-hospital paediatric case fatality rate (*by common paediatric conditions*)	% of children who were diagnosed with Sepsis, Pneumonia, Malaria, Meningitis or Severe Acute Malnutrition (SAM) and died in the health facility	Outcome & impact	Inpatient	% of children who were diagnosed with Sepsis, Pneumonia, Malaria, Meningitis or SAM and died in the health facility *(Includes deaths in the emergency ward but does not include children who died upon arrival at the hospital, child deaths during outpatient visits, and institutional neonatal deaths)*	# of children who visited the health facility and were diagnosed with Sepsis, Pneumonia, Malaria, Meningitis or SAM during reporting period	•Conditions •Levels of health facilities (e.g. secondary level) •Age groups (0–7 days, 8–27, 28–59 days, 60 days- < 1 year, 1- < 5 y, 5- < 10, 10- < 15 y) •Death within and after 24 h of admission to the facility •Sex	Routine HMIS	Review of paediatric ward register or patient medical records, paediatric death audit and triangulation of information if possible	Monthly
3.Essential IMNCI^b^ assessment for the sick child	% of sick < 5 children who were assessed in the health facility based on key IMNCI assessment criteria	Process / Output	Inpatient & Outpatient	# of sick < 5 children who were assessed based on key IMNCI assessment criteria^c^	# of sick < 5 children who visited the health facility during the reporting period	•Facility type (e.g. health center) •IMNCI assessment components •Sex •Age (0—< 2 month, 2 month—< 5 years)	Patient medical records	Review of medical records, periodic health facility assessments, quality-of-care assessments, etc	Monthly
4.Treatment of PSBI^d^ at outpatient level	% of young infants classified as having PSBI or signs of PSBI, or very severe disease or sepsis—who were prescribed appropriate antibiotics according to WHO guidelines	Process / Output	Outpatient	# of young infants (< 2 months of age) classified as having PSBI or any child with related signs or very severe disease or sepsis, who were prescribed appropriate antibiotics according to WHO guidelines	# of sick young infants (< 2 months of age) classified as having PSBI or any child with related signs or very severe disease or sepsis who visited health facility during reporting period	•Sex •Weight cutoffs (< 2000 g, ≥ 2000 g)	Patient medical records	Review of medical records, periodic health facility assessments, quality-of-care assessments, etc	Monthly
5.KMC^e^ initiation for infants weighing 2000 g or less	% of infants initiated on KMC	Process / Output	Inpatient & Outpatient	# of infants weighing 2000 g or less who were initiated on KMC	# of infants weighing 2000 g or less who were born in or presented to the health facility during the reporting period	•Facility type (e.g. health center) •Sex •Weight (≤ 1500 g and 1500—< 2000 g) •Immediate/non-immediate	Routine HMIS	Review of ward registers or patient medical records	Monthly
6.Pneumonia treatment with 1st choice antibiotic	% of children aged between 7 days and 5 years who were prescribed amoxicillin for treatment of pneumonia	Process / Output	Inpatient & Outpatient	# of children aged between 7 days and 5 years who were diagnosed with pneumonia or showed signs of fast breathing and/or chest indrawing and were prescribed oral amoxicillin	# of children aged between 7 days and 5 years seen in the same health facility and period with pneumonia or fast breathing and/or chest indrawing who visited the health facility during the reporting period	•Sex •Facility type •Age (7–59 days, 2 month—< 5 years)	Routine HMIS	Review of ward registers or patient medical records	Monthly
7.Management of acute watery diarrhea among children < 5 years old	% of children < 5 years diagnosed with acute watery diarrhea in a health facility who received appropriate treatment for diarrhea	Process / Output	Inpatient & Outpatient	# of children < 5 years who were diagnosed with acute watery diarrhea and received ORS and Zinc supplementation (if 2 months- < 5 years)	# of children < 5 years old with diagnosis of acute watery diarrhea who visited health facility during the reporting period	•Age (0–59 days, 2 months- < 5 years) •Sex •Facility type	Routine HMIS	Review of ward registers or patient medical records	Monthly
8.Paediatric malaria diagnostic testing rate in malaria endemic areas^f^	% of children < 15 years old in malaria endemic areas who presented to the health facility with fever and their malaria test results are available	Process / Output	Inpatient & Outpatient	# of children < 15 years old in malaria endemic areas who presented to the health facility with fever for whom malaria test results are available (*results from microscopy or malaria Rapid Diagnostic Test*)	# of children < 15 years old in malaria endemic areas who visited health facility with fever during reporting period	•Facility type •Sex •Diagnosis •Age (< 5 years old, 5- < 10 years old, 10—< 15 years old)	Routine HMIS	Review of ward registers or patient medical records	Monthly
9.Treatment of uncomplicated SAM^g^	% of children aged between 6 months and 5 years with uncomplicated SAM who were treated according to WHO guidelines	Process / Output	Outpatient	# of children aged between 6 month and 5 years with uncomplicated SAM who received oral amoxicillin and RUTF^h^	# of children aged between 6 month and 5 years diagnosed with uncomplicated SAM who visited health facility during reporting period	•Sex	Routine HMIS	Review of ward registers or patient medical records	Monthly
10.Management of anemia	% of children aged between 2 months up to 15 years with anemia who were treated according to WHO guidelines	Process / Output	Inpatient & Outpatient	# of children aged between 2 months and 15 years diagnosed with who received the correct prescription for anemia according to WHO guidelines^i^	# of children aged between 2 months and 15 years diagnosed with anemia who visited health facility during reporting period	•Sex •Age groups (< 1 year, 1—< 5, 5—< 15)	Routine HMIS	Review of ward registers or patient medical records	Monthly
11.HIV testing for the mother and/or the child (in high HIV prevalence settings)	% of children < 2 years old for whom the HIV status of the mother and/or the child are known	Process / Output	Inpatient & Outpatient	# of children < 2 years old for whom the HIV status of the mother and/or the child are known (positive or negative)	# of children < 2 years old who visited the health facility during reporting period	•Sex of the child •Mother or child	Routine HMIS	Review of ward registers or patient medical records	Monthly
12.TB evaluation for children with presumptive TB	% of children < 15 years eligible for TB screening, who were referred or further assessed for TB	Process / Output	Inpatient & Outpatient	# of children who reported a cough duration > 14 days or were diagnosed with SAM or had confirmed HIV infection, who were referred or further assessed for TB	# of children with SAM or confirmed HIV infection or cough duration > 14 days who visited health facility during reporting period	•Sex •Age groups (< 1 year, 1—< 5, 5—< 15)	Routine HMIS	Review of ward registers or patient medical records	Monthly
13.Missed-Opportunity for vaccination (MOV)	% of children < 2 years of age eligible for DTP-Hep-B-HIB, IPV, RTV, PCV or Measles-containing vaccine, who were administered all catch up immunization during medical visits	Process / Output	Inpatient & Outpatient	# of children < 2 years of age eligible for DTP-Hep-B-HIB, IPV, RTV, PCV and Measles-containing vaccine (unvaccinated or partially vaccinated with these vaccines according to their age and national immunization schedule) who were administered all catch up immunization with DTP-Hep-B-HIB, IPV or Measles-containing vaccines during medical visits	# of children < 2 years of age eligible for DTP-Hep-B-HIB, IPV, RTV, PCV and Measles-containing vaccine (unvaccinated or partially vaccinated with these vaccines according to their age and national immunization schedule) who received medical care in the health facility during reporting period	•Sex •Age (< 1 year, 1- < 2 years) •By antigens	Routine HMIS	RHIS, facility registry, medical record review, caregiver exit interview or observation with concurrent review of child’s vaccination card	Monthly
14.Inappropriate use of antibiotic for cough or cold	% of children with only cough or cold to whom an antibiotic was prescribed	Process	Inpatient & Outpatient	# of children in a health facility with only cough or cold or any of the following unspecified RTI diagnosis (No pneumonia, RTI, URTI) and no comorbidity requiring antibiotic treatment (E.g., Pneumonia, Severe Pneumonia, SAM, Very Severe Disease, Sepsis, Meningitis, Dysentery, Cholera, HIV +) to whom an antibiotic was prescribed	# of children with only cough or cold or any of the following unspecified RTI diagnosis (No pneumonia, RTI, URTI) and no comorbidity requiring antibiotic treatment (E.g., Pneumonia, Severe Pneumonia, SAM, Very Severe Disease, Sepsis, Meningitis, Dysentery, Cholera, HIV +) who visited health facility during reporting period	•Facility types •Sex •Diagnosis	Routine HMIS	Review of ward registers or patient medical records	Monthly
15.Completion of medical documentation	% of medical records of children who received care in the health facility during the reporting period with complete key patient information	Input	Inpatient & Outpatient	# of paediatric medical records (or registry entries) with complete key patient information *(patient demographics (age, sex), assessment findings, classification / diagnosis, treatment, counselling, and care outcomes*)	# of paediatric medical records (or registry entries) of children < 15 years who visited health facility during reporting period	•Service level (inpatient, outpatient)	Facility registry or medical records	Facility registry or medical record review	Monthly
16.Paediatric QoC indicator review	% of health facilities that have conducted monthly paediatric QoC indicator data review during the last 3 months	Process / Output	Inpatient & Outpatient	# of health facilities that have conducted monthly QoC indicator data review during the last 3 months	# of facilities assessed during the reporting period	•Type of health facilities	Survey	Periodic health facility survey: review of facility documentation	Quarterly
17.Patient knowledge and understanding of their condition and treatment plan	% of children < 15 years old (or their caregivers) who can describe the child’s condition and how to take the treatment at home	Process / Output	Inpatient & Outpatient	# of children < 15 years old (or their caregivers) who can describe the child’s condition and how to take the treatment at home	# of children (or their caregivers) who were discharged from the health facility and were interviewed during reporting period	•Child vs caregiver •Service level (inpatient, outpatient) •Type of health facility	Survey or interview records	Client Exit interview	Quarterly
18.Satisfaction with decision-making process for care	% of children < 15 years old (or their caregivers) who are satisfied with the decision-making process for care	Outcome (patient-reported)	Inpatient & Outpatient	# of children < 15 years old (or their caregivers) who reported being satisfied with the decision-making process for care	# of children < 15 years old who received care or their caregivers who were interviewed in health facility during reporting period	•Type of health facilities •Child vs caregiver •Age of child •Health condition	Survey or interview records	Facility Survey, of client exit interviews	Quarterly
19.Pre-discharge counselling on danger signs and feeding during illness	% of caregivers of children < 5 years who are aware of the danger signs for pediatric illness, when to seek care and how to feed their children during the illness	Process / Output	Inpatient & Outpatient	# of caregivers of children < 5 years who reported being aware of the danger signs of their children, when to seek care and how to feed their children during the illness (e.g. giving extra fluids and continue feeding)	# of caregivers of children < 5 years who received care and were interviewed in health facility during reporting period	•Type of health facility	Facility Survey, of client exit interviews	Periodic health facility survey: review of facility documentation	Quarterly
20.Awareness of child rights during health care	% of children < 15 years old (or their caregivers) who reported being adequately informed about their rights to care	Process / Output	Inpatient & Outpatient	# of children < 15 years old (or their caregivers) who reported being adequately informed about their rights to care (e.g. free treatment, medication, food, bedding, room-in etc.)	# of children < 15 years old (or their caregivers) who received care and were interviewed in health facility during reporting period	•Age < 2 months, 2- < 5 years, 5- < 15 years, •Type of health facility	Facility Survey, of client exit interviews	Periodic health facility survey: review of facility documentation	Quarterly
21.Disrespectful care for the child or caregiver	% of children < 15 years old (or their caregivers) who reported being mistreated during care	Outcome (patient-reported)	Inpatient & Outpatient	# of children < 15 years old (or their caregivers) who reported being mistreated during care *(Includes those who felt that they were being yelled at or screamed at (verbal abuse), or were hit, or pinched (physical abuse)*	# of children < 15 years old (or their caregivers) who received care and were interviewed in health facility during reporting period	•Age < 5 years, 5- < 15 years •Type of health facility •Type of mistreatment •Child vs caregiver	Survey or interview records	Facility Survey, of client exit interviews	Quarterly
22.Accompaniment during care	% of children < 15 years old whose caregivers were able to stay with them during minor medical procedures	Process/ output	Inpatient & Outpatient	# of children < 15 years old whose caregivers were able to stay with them during medical procedures	# of children < 15 years old (or their caregivers) who received care and were interviewed in health facility during reporting period	•Type of health facility	Survey or interview records	Periodic health facility survey:	Quarterly
23.Access to play and educational material during hospitalization	% of children^j^ (or their caregivers) who reported that the child was able to play and access educational materials during hospitalization	Input	Inpatient	# of children (or their caregivers) who reported that the child was able to play and access educational material during hospitalization	# of children treated as inpatient or their caregiver who were interviewed in health facility during reporting period	•Age 5- < 10, 10- < 15 years •Type of health facility	Survey or interview records	Periodic health facility survey	Quarterly
24.Clinical mentorship or training	% of health workers providing care for children who received clinical mentorship or training in the past 6 months	Input	Inpatient & Outpatient	# of childcare providers who had reported interactions with professional mentors or participated in continuous professional development to ensure clinical competence and improve performance in the past 6 months	# of childcare providers working in the health facility who were interviewed during reporting period	•Provider cadre, •Facility type	Provider interviews	Facility Survey: Provider interviews	Quarterly
25.Stock out of essential child health medicines	# of days in the past 3 months when there were stock outs of at least 3 essential children medicines	Input	Inpatient & Outpatient	Total number of days with stock outs of at least three^k^ or more essential medicines. For outpatient facilities, essential medicines include: 19 medications; for inpatient facilities, essential medications include 9 additional essential medications	N/A	•Types of medications, •Inpatient/outpatient	Inventory review or facility-in charge interview	Inventory	Quarterly

^a^ Health Management Information System.

^b^ Integrated Management of Newborn and Childhood Illness.

^c^ Presence or absence of danger signs (ability to drink or breastfeed; vomits everything; convulsions, lethargy) and received rapid physical and clinical assessment including, weight, Z-score or MUAC, respiratory rate, temperature, cough, difficult breathing/chest indrawing, diarrhea/dehydration status, vaccination status, and palmar pallor.

^d^ Possible Severe Bacterial Infection.

^e^ KMC: Kangaroo Mother Care (defined as care of small infants weighing 2000 g or less carried skin-to-skin with the mother according to WHO guidelines).

^f^ According to WHO, malaria endemic areas are area in which there is an ongoing, measurable incidence of malaria infection and mosquito-borne transmission over a succession of years. These countries include most Sub-Saharan countries, excluding South Africa and eSwatini, but including Pakistan, Afghanistan, India, Papua New Guinea, and Indonesia.

^g^ Severe Acute Malnutrition.

^h^ Ready-to-Use-Therapeutic Food.

^i^ Iron and mebendazole (if 1 year or older and not given mebendazole last 6 months).

^j^ School-age limits will be aligned to specific country’s context.

^k^ Amoxicillin, injectable gentamicin, and zinc3.

The final recommended set of core indicators are not evenly distributed across QS, MD, system categories, and service levels. Most of them are process-related (64%) followed by outcome/impact indicators (20%). A large proportion of the core indicators (84%) can be measured both at outpatient and inpatient levels. Fourteen core indicators were related to MD-1 to measure prioritized clinical content areas along with unnecessary, harmful practices, patient safety, and rational use of medications. The indicator “Institutional Child Mortality Rate” as an impact indicator cuts across different MD and QS. For simplicity, this indicator was mapped to MD-1. Four core indicators were mapped to MD-2, two of which relate to QS-2, and the other two on QS 7&8. Lastly, seven core indicators reflected MD-3 on child- and family-centred practices and experiences of care. Three of these were mapped to QS-4 while the other two were mapped to QS-5. The distribution of the parent QI catalogue indicators across different measurement typologies is presented in the Additional File 4.

## Discussion

Paediatric QoC measurement has long been a complex area with limited global guidance and recommendations on how to develop paediatric QoC indicators. In many settings, there are general challenges of measuring QoC, including those related to data sources and quality. However, there are other specific challenges of measuring QoC among children due to their different needs and capabilities. At a minimum, the well balanced set of paediatric QoC indicators should be balanced to include measures of preventive care; treatment for acute conditions; treatment for diseases and disabilities requiring long term care; as well as different indicator typologies including input, process, and outcome indicators. Furthermore, paediatric QoC indicators should include measures of experience of care from the child and the caregiver perspective.

In response to the current gaps in normative guidance around paediatric QoC measurement, we make recommendations for a core set of QoC indicators for measuring and monitoring paediatric QoC in health facilities, and reporting at all levels for learning, accountability, as well as progress tracking.

### The recommended indicators in context

We make recommendations for core indicators that are mapped to the paediatric QSs for health facilities. Almost all QSs are represented by at least one core indicator. However, certain QSs have greater representation than others because they are more complex in terms of the number of quality dimensions to be considered and their relative importance in influencing health care outcomes. This approach resulted in prioritization of more provision and experience of care indicators, compared to indicators that measure availability of inputs. While important, inputs alone do not guarantee that the service is provided correctly and consistently.

Our goal was to develop a parsimonious set of robust indicators that provide high level insights into the quality of paediatric care in health facilities, as opposed to a comprehensive suite of indicators that provide detailed measurement of the quality of paediatric care. Less emphasis was thus placed on overall feasibility of measurement in the short term, especially in LMICs, mainly because of two reasons.

Firstly, QoC measurement is a relatively new area of measurement in most LMICs. Many countries in such settings are yet to institutionalise QoC measurement within their mainstream health information systems [18]. Developing paediatric QoC indicators with strong emphasis on feasibility of measurement would have yielded an even more imbalanced set of indicators, from the perspective of their ability to measure the QSs from which they were derived, and the indicator metadata that defines critical attributes for QoC indicators. We therefore recognize that not all the recommended core indicators can be measured across all settings in the short-term. Globally, the ability of countries and institutions to measure, collect, report and use data for most health care indicators in general varies greatly across countries, including in high income settings [19–21]. Our argument is therefore that the audience for these indicators—which includes countries and partners working in the paediatric QoC programming and QI space – should, in the short term, select and use the recommended indicators for which data are available in their health information systems. Users can then continue to improve their data systems to accommodate indicators that are not currently reported in standardized medical documentation, or are more complex to measure, or for which more data elements are required to collect the full set of core indicators. We also note that even in high-income settings, introducing new indicators in hospital settings may come with administrative challenges. The existing health information systems may require some changes such as the adaptation of patient administration forms and facility registers, and an increase in the administrative burden of data collection, collation and reporting [22].

Secondly, the recommended set is a response to urgent programmatic needs on a global scale in paediatric QoC implementation and measurement. Field-testing of indicators takes time and resources and there are methodological weakness of assessing the feasibility and sustainability of measuring and monitoring newly-developed indicators outside research contexts [23, 24]. Field-testing of these indicators is the next step that WHO and its partners will lead in multiple countries.

### Strength and limitations

The implementation of expert recommendations on the methodology to develop the core indicators was not without challenges. The health status and risk factors for illness for young infants, infants, children under 5, and young adolescents differ and change over time. This created some challenges in prioritizing core indicators that are reflective of all age categories. The age categories where quality measurement is most crucial were thus prioritized, which introduced some imbalances. Furthermore, the indicators recommended for measuring experience of care will likely require the collection of views of children and/or their caregivers, as appropriate, and the content of the information provided would depend on several individual and contextual factors, including the age of the child and local cultural norms regarding care, age of majority, and parental rights. For example, meaningful participation and input from children can vary based on age and condition. Similarly, child health rights, especially young adolescents, may vary from one country to another which would render global reporting a challenge. In some cases, the experience of the caregiver and the child may be different and future work will be needed to understand how to measure these both together and separately.

A systematic review of methods used to develop quality indicators showed that patient participation during development of quality of care indicators remains uncommon and there is no standardized methodology on how best to involve patients and communities in the process [27]. To address this limitation, organizations and experts working on paediatric experience of care and patient care advocacy and child rights were included in development of experience of care standards and the indicator prioritization processes. Furthermore, during the field-based evaluation of the proposed indicators, WHO will seek to involve children and their caregivers to test the smaller set of indicators that focus on respectful and experience of care in various settings.

Some clinical indicators are compound metrics which require computation using several data elements, in some instances from different data sources. This was inevitable given the focus on robust measurement of quality as opposed to a convenient approach. While compound indicators provide high level, comprehensive understanding of the QoC at the national and global levels, these indicators need further disaggregation or additional catalogue indicators at the subnational and facility levels to identify and address the root-causes of poor quality services.

Furthermore, we acknowledge that the indicator prioritization algorithm we used was complex, and in some places, the algorithm involved the use of subjective criteria due to lack of scientific evidence to guide the process. For example, the criteria used to prioritize some input measures involved weights which are arguably subjective.

Similarly, because of the goal of keeping the list of core indicators short, there are few areas (e.g., referral systems) that are not included in the core list but could be part of a future research agenda. Despite all these challenges, however, the iterative peer-review process with various expert groups added value to the robustness of the proposed suite of indicators. Furthermore, the criteria used to prioritize the indicators are evidence-based and presented an opportunity to select and define indicators that are core for paediatric QoC measurement in health facilities where the QSs are supposed to be implemented.

Lastly, the excel tool which we developed to operationalize the indicator prioritization methodology, allows the user to add additional indicators, change assigned scores and prioritization weights, and designate cut-off scores. After this, the tool automatically selects the core and catalogue indicators. These features, along with the ability to systematize and sort the measures by various categories, are particularly important in the context of constantly changing evidence and emerging local or global priorities.

### Implications for research and practice

The proposed suite of indicators and their metadata are part of WHO’s living normative guidance on MNCH QoC measurement. They are not a final product and may be refined or revised based on the findings from future research on their validity in various contexts, especially in LMICs. An important next step will therefore be a large-scale, multicounty study to determine how these indicators can best be measured in different contexts. Immediate utilization of these indicators in practice and on a global scale will require a tiered approach that considers varying country contexts. Countries will need to decide which indicators they can measure and report on in the short-term, and which ones require further health information system adaptation in the medium to long terms.

## Conclusions

The deductive, multistep approach we used to develop core paediatric QoC indicators yielded a robust set of indicators that represent all the 8 QSs. The recommended indicators can be adopted at country level using a tiered approach to support urgent paediatric and young adolescent quality-of-care programming and improvement work. However, there is a need for further research to assess the feasibility of their measurement across settings as well as standardization and validation for global reporting in future.

## Disclaimer

The authors alone are responsible for the views expressed in this article and they do not necessarily represent the views, decisions, or policies of the institutions with which they are affiliated.

## Supplementary Information

**Additional file 1: Supplementary file 1.** Summary of standards and quality statements

**Additional file 2: Supplementary file 2.** Prioritization algorithm

**Additional file 3: Supplementary file 3.** Listof prioritized clinical content areas for the selection of measures

**Additional file 4: Supplementary file 4.** Distributionof QI catalogue indicators by MDs, QSs, indicator classification, and servicelevel

## Data Availability

All data generated or analysed during this study are included in this published article [and its supplementary information files].

## Abbreviations

LMICs: Low- and middle-income countries
WHO: World Health Organization
QSs: Quality Standards
MNH: Maternal and Newborn Health
QoC: Quality of Care
QDs: Quality Domains
QMs: Quality Measures
MNCH: Maternal, Newborn, and Child Health
QI: Quality Improvement
CHAT: Child Health Accountability Tracking
USAID: United States Agency for International Development
UNICEF: United Nations Children's Fund
SL: Service Level
SC: System Category
CA: Clinical Area
MD: Measurement Domain
MSD: Measurement Sub-Domain
