# Supplementary material for: Global core indicators for measuring WHO’s paediatric quality-of-care standards in health facilities: development and expert consensus
Source: BMC Health Serv Res. 2022 Jul 8;22:887. doi: 10.1186/s12913-022-08234-5 (PMC9270792; doi:10.1186/s12913-022-08234-5)
Supplement: Supplementary file 1 — Additional file 1: Supplementary file 1. Summary of standards and quality statements [file 12913_2022_8234_MOESM1_ESM.docx]

Appendix 1

Summary of standards and quality statements

These standards for the quality of paediatric care in health facilities form part of normative guidance for improving the quality of maternal, newborn, child and adolescent health care. In view of the importance of the continuum of both the life-course and service delivery, these standards build on the Standards for improving the quality of maternal and newborn care in health facilities during labour, childbirth and the early postnatal period. They are derived from WHO Guidelines Review Committee approved recommendations, guidelines and international best practices in care of children and young adolescents.

**STANDARD 1.**

Every child receives evidence-based care and management of illness according to WHO guidelines

**Quality statement 1.1**

All children are triaged and promptly assessed for emergency and priority signs to determine whether they require resuscitation and receive appropriate care according to WHO guidelines.

**Quality statement 1.2**

All sick infants, especially small newborns, are thoroughly assessed for serious bacterial infection and receive appropriate care according to WHO guidelines.

**Quality statement 1.3**

All children with cough or difficult breathing are correctly assessed, classified and investigated and receive appropriate care and/or antibiotics for pneumonia, according to WHO guidelines.

**A Quality statement 1.4**

ll children with diarrhoea are correctly assessed and classified and receive appropriate rehydration and care, including continued feeding, according to WHO guidelines.

**Quality statement 1.5**

All children with fever are correctly assessed, classified and investigated and receive appropriate care according to WHO guidelines.

**Quality statement 1.6**

All infants and young children are assessed for growth, breastfeeding and nutrition, and their carers receive appropriate support and counselling, according to WHO guidelines.

**Quality statement 1.7**

All children at risk for acute malnutrition and anaemia are correctly assessed and classified and receive appropriate care according to WHO guidelines.

**Quality statement 1.8**

All children at risk for tuberculosis (TB) and/or HIV infection are correctly assessed and investigated and receive appropriate management according to WHO guidelines.

**Quality statement 1.9**

All children are assessed and checked for immunization status and receive appropriate vaccinations according to the guidelines of the WHO expanded programme on immunization.

**Quality statement 1.10**

All children with chronic conditions receive appropriate care, and they and their families are sufficiently informed about their condition(s) and are supported to optimize their health, development and quality of life.

**Quality statement 1.11**

All children are screened for evidence of maltreatment, including neglect and violence, and receive appropriate care.

**Quality statement 1.12**

All children with surgical conditions are screened for surgical emergencies and injury and receive appropriate surgical care.

**Quality statement 1.13**

All sick children, especially those who are most seriously ill, are adequately monitored, reassessed periodically and receive supportive care according to WHO guidelines.

**Quality statement 1.14**

All children receive care with standard precautions to prevent health care- associated infections.

**Quality statement 1.15**

All children are protected from unnecessary or harmful practices during their care.

**STANDARD 2.**

The health information system ensures the collection, analysis and use of data to ensure early, appropriate action to improve the care of every child.

**Quality statement 2.1**

Every child has a complete, accurate, standardized, up-to-date medical record, which is accessible throughout their care, on discharge and on follow-up.

**Quality statement 2.2**

Every health facility has a functional mechanism for data collection, analysis and use as part of its activities for monitoring performance and quality improvement .

**Quality statement 2.3**

Every health facility has a mechanism for collecting, analysing and providing feedback on the services provided and the perception of children and their families on the care received.

**STANDARD 3.**

Every child with condition(s) that cannot be managed effectively with the available resources receives appropriate, timely referral, with seamless continuity of care.

**Quality statement 3.1**

Every child who requires referral receives appropriate prereferral care, and the decision to refer is made without delay.

**Quality statement 3.2**

Every child who requires referral receives seamless, coordinated care and referral according to a plan that ensures timeliness.

**Quality statement 3.3**

For every child referred or counter-referred within or among health facilities, there is appropriate information exchange and feedback to relevant health care staff.

**STANDARD 4.**

Communication with children and their families is effective, with meaningful participation, and responds to their needs and preferences.

**Quality statement 4.1**

All children and their carers are given information about the child’s illness and care effectively, so that they understand and cope with the condition and the necessary treatment.

**Quality statement 4.2**

All children and their carers experience coordinated care, with clear, accurate information exchange among relevant health and social care professionals and other staff.

**Quality statement 4.3**

All children and their carers are enabled to participate actively in the child’s care, in decision-making, in exercising the right to informed consent and in making choices, in accordance with their evolving capacity.

**Quality statement 4.4**

All children and their carers receive appropriate counselling and health education, according to their capacity, about the current illness and promotion of the child’s health and well-being.

**STANDARD 5.**

Every child’s rights are respected, protected and fulfilled at all times during care, without discrimination.

**Quality statement 5.1**

All children have the right to access health care services, with no discrimination of any kind.

**Quality statement 5.2**

All children and their carers are made aware of and given information about children’s rights to health and health care.

**Quality statement 5.3**

All children and their carers are treated with respect and dignity, and their right to privacy and confidentiality is respected.

**Quality statement 5.4**

All children are protected from any violation of their human rights, physical or mental violence, injury, abuse, neglect or any other form of maltreatment.

**Quality statement 5.5**

All children have access to safe, adequate nutrition that is appropriate for both their age and their health condition during their care in a facility.

**STANDARD 6.**

All children and their families are provided with educational, emotional and psychosocial support that is sensitive to their needs and strengthens their capability.

**Quality statement 6.1**

All children are allowed to be with their carers, and the role of carers is recognized and supported at all times during care, including rooming-in during the child’s hospitalization.

**Quality statement 6.2**

All children and their families are given emotional support that is sensitive to their needs, with opportunities for play and learning that stimulate and strengthen their capability.

**Quality statement 6.3**

Every child is assessed routinely for pain or symptoms of distress and receives appropriate management according to WHO guidelines.

**STANDARD 7.**

For every child, competent, motivated, empathic staff are consistently available to provide routine care and management of common childhood illnesses.

**Quality statement 7.1**

All children and their families have access at all times to sufficient health professionals and support staff for routine care and management of childhood illnesses.

**Quality statement 7.2**

Health professionals and support staff have the appropriate skills to fulfil the health, psychological, developmental, communication and cultural needs of children.

**Quality statement 7.3**

Every health facility has managerial leadership that collectively develops, implements and monitors appropriate policies and legal entitlements that foster an environment for continuous quality improvement.

**STANDARD 8.**

The health facility has an appropriate, child-friendly physical environment, with adequate water, sanitation, waste management, energy supply, medicines, medical supplies and equipment for routine care and management of common childhood illnesses.

**Quality statement 8.1**

Children are cared for in a well-maintained, safe, secure physical environment with an adequate energy supply and which is appropriately designed, furnished and decorated to meet their needs, preferences and developmental age.

**Quality statement 8.2**

Child-friendly water, sanitation, hand hygiene and waste disposal facilities are easily accessible, functional, reliable, safe and sufficient to meet the needs of children, their carers and staff.

**Quality statement 8.3**

Child-friendly, age-appropriate equipment designed to meet children’s needs in medical care, learning, recreation and play are available at all times.

**Quality statement 8.4**

Adequate stocks of child-friendly medicines and medical supplies are available for the routine care and management of acute and chronic childhood illnesses and conditions.
