# Supplementary material for: Global core indicators for measuring WHO’s paediatric quality-of-care standards in health facilities: development and expert consensus
Source: BMC Health Serv Res. 2022 Jul 8;22:887. doi: 10.1186/s12913-022-08234-5 (PMC9270792; doi:10.1186/s12913-022-08234-5)
Supplement: Supplementary file 3 — Additional file 3: Supplementary file 3. Listof prioritized clinical content areas for the selection of measures [file 12913_2022_8234_MOESM3_ESM.docx]

***Additional File 3*** List of prioritized clinical content areas for the selection of measures

| Routine care of every child: nutrition/feeding assessment, growth monitoring and counselling |
| --- |
| Acute malnutrition and Anaemia: assessment, classification, and treatment |
| TB and HIV |
| Routine care of every child: Vaccination and Vitamin A assessment and provision |
| Children with chronic conditions (Asthma, Diabetes, cardiac disease, epilepsy, sickle cell disease, cerebral palsy) |
| Surgical care (assessment and treatment) |
| Supportive care of small and sick newborns |
| Cross-cutting IPC and WASH |
| Cross-cutting: Prevention of unnecessary and harmful practices |
| Other Infectious diseases and local infections |
| Integrated Management of Newborn and Childhood Illness |
