# Supplementary material for: Global core indicators for measuring WHO’s paediatric quality-of-care standards in health facilities: development and expert consensus
Source: BMC Health Serv Res. 2022 Jul 8;22:887. doi: 10.1186/s12913-022-08234-5 (PMC9270792; doi:10.1186/s12913-022-08234-5)
Supplement: Supplementary file 4 — Additional file 4: Supplementary file 4. Distributionof QI catalogue indicators by MDs, QSs, indicator classification, and servicelevel [file 12913_2022_8234_MOESM4_ESM.docx]

***Additional File 4:***  Distribution of QI catalogue indicators by MDs, QSs, indicator classification, and service level

| **Measurement domains & Standards** | | **Classification** | | | | | | **Service level** | | | | | |  |  |
| --- | --- | --- | --- | --- | --- | --- | --- | --- | --- | --- | --- | --- | --- | --- | --- |
|  |  | **Input** | | **Process** | | **Outcome / Impact** | | **Inpatient** | | **Outpatient** | | **Both** | | **Total** | |
|  |  | n | (%) | n | (%) | n | (%) | n | (%) | n | (%) | n | (%) | n | (%) |
| **MD-1:** Evidence-based practices for routine care of children and management of illness | | 24 | (14) | 38 | (22) | 24 | (14) | 24 | (14) | 13 | (8) | 49 | (28) | 86 | (50) |
|  | Standard 1: Evidence-based practices for routine care of children and management of illnesses | 24 | (14) | 38 | (22) | 24 | (14) | 24 | (14) | 13 | (8) | 49 | (28) | 86 | (50) |
| **MD-2:** Cross-cutting supporting facility level health systems | | 36* | (21) | 6 | (3) | 4 | (2) | 5 | (3) | 1 | (1) | 40* | (23) | 46 | (27) |
|  | Standard 2: Actionable information systems | 11 | (6) | 0 | (0) | 0 | (0) | 1 | (1) | 0 | (0) | 13 | (8) | 11 | (6) |
|  | Standard 3: Functioning referral systems | 5 | (3) | 2 | (1) | 2 | (1) | 1 | (1) | 1 | (1) | 7 | (4) | 9 | (5) |
|  | Standard 7: Competent, motivated, empathetic human resources | 5 | (3) | 3 | (2) | 1 | (1) | 0 | (0) | 0 | (0) | 9 | (5) | 9 | (5) |
|  | Standard 8: Essential child and adolescent-friendly physical resources | 13 | (8) | 1 | (1) | 1 | (1) | 3 | (2) | 0 | (0) | 12 | (7) | 15 | (9) |
| **MD-3**: Child and family-centered practices/experience of care | | 15 | (9) | 6 | (3) | 19 | (11) | 14 | (8) | 1 | (1) | 25 | (15) | 40 | (23) |
|  | Standard 4: Effective communication and meaningful participation | 3 | (2) | 3 | (2) | 7 | (4) | 5 | (3) | 1 | (1) | 7 | (4) | 13 | (8) |
|  | Standard 5: Respect, protection, and fulfilment of child rights | 9 | (5) | 1 | (1) | 6 | (3) | 3 | (2) | 0 | (0) | 13 | (8) | 16 | (9) |
|  | Standard 6: Emotional and psychological support | 3 | (2) | 2 | (1) | 6 | (3) | 6 | (3) | 0 | (0) | 5 | (3) | 11 | (6) |
| **Total # of catalogue indicators** | | **75** | **(44)** | **50** | **(29)** | **47** | **(27)** | **43** | **(25)** | **15** | **(9)** | **114** | **(66)** | **172** | **(100)** |
